# Serological and Molecular Detection of Citrus Tristeza Virus: A Review

**DOI:** 10.3390/microorganisms12081539

**Published:** 2024-07-27

**Authors:** Pengxiang Shang, Longfa Xu, Tong Cheng

**Affiliations:** 1 State Key Laboratory of Vaccines for Infectious Diseases, Xiang An Biomedicine Laboratory, School of Life Sciences, School of Public Health, Xiamen University, Xiamen 361102, China; pengxiangshang@xmu.edu.cn; 2 National Institute of Diagnostics and Vaccine Development in Infectious Diseases, Xiamen University, Xiamen 361102, China

**Keywords:** *Citrus tristeza virus*, CTV, detection, serology, molecular detection

## Abstract

*Citrus tristeza virus* (CTV) is a globally pervasive and economically significant virus that negatively impacts citrus trees, leading to substantial reductions in fruit yield. CTV occurs within the phloem of infected plants, causing a range of disease phenotypes, such as stem pitting (SP), quick decline (QD), and other detrimental diseases. Research on CTV is challenging due to the large size of its RNA genome and the diversity of CTV populations. Comparative genomic analyses have uncovered genetic diversity in multiple regions of CTV isolates’ genomes, facilitating the classification of the virus into distinct genotypes. Despite these challenges, notable advancements have been made in identifying and controlling CTV strains through serological and molecular methods. The following review concentrates on the techniques of nucleic acid identification and serological analysis for various CTV isolates, assisting in the comparison and evaluation of various detection methods, which are crucial for the effective management of CTV diseases, and so contributes to the innovation and development of CTV detection methods.

## 1. Introduction

Citrus constitutes the world’s most extensively cultivated fruit crop, leading globally both in terms of planting area and yield. CTV is one of the most destructive and widely distributed pathogens affecting citrus, posing a serious threat to the global citrus industry because of its significant negative impact on tree vitality and fruit yield, even leading to tree death in some cases. Depending on the various hosts and viral strains, CTV may cause different symptoms, including seedling yellows (SY), rapid decline, and SP [1,2]. Sour orange rootstock is highly sensitive to CTV, and sweet orange grafted on sour orange will die if infected with a QD strain. Over the last century, due to the prevalence of QD strains in regions such as Argentina (1931), Brazil (1937), and America (1951), over 100 million citrus trees grafted onto sour orange rootstock have died [3,4]. Furthermore, CTV infection can significantly degrade the quality of citrus fruit, facilitating lower market values and substantial economic losses for growers. At present, no curative treatments for CTV are available, leading to the death or removal of infected trees. In some instances, infection by mild strains of CTV may only cause leaf yellowing in citrus trees, which generally does not significantly affect fruit yield [3]. To mitigate CTV-related losses, citrus producers can adopt preventive measures, such as propagating disease-free seedlings, conducting consistent surveillance of CTV within orchards, and promptly managing vector populations.

## 2. Pathogen and Symptomatology

CTV, a plant pathogenic virus that infects citrus trees, was first reported in Brazil and Argentina in the 1930s [5]. This pathogen belongs to the family *Closteroviridae* and genus *Closterovirus*. CTV exhibits a highly restricted host range, predominantly affecting species in the Rutaceae family [4]. Genetics research has helped to identify 11 genetic lineages of CTV globally: T3, T30, T36, VT, S1, HA16-5, RB, T68, A18, M1, and L1 genotypes [6]. CTV possesses an approximately 19.3 kilobase (kb) genome. It has the largest non-segmented RNA genome, which is one of the most intricate among plant viruses identified thus far [4,7]. The genome of this virus comprises 12 open reading frames (ORFs) that encode for more than 19 protein products. ORF1a and ORF1b translate into a polyprotein essential for viral replication initiated from the 5′ end of the genomic RNA. This polyprotein is characterized by two papain-like cysteine protease domains (L1 and L2), a methyltransferase-like domain (MT-like), a helicase-like domain (HEL-like), and an RNA-dependent RNA polymerase (RdRp) domain [8,9,10]. The proteins encoded by ORFs 2–11 have been identified as p33, p6, p65 (comparable to the 70 kDa heat shock protein HSP70), p61, p27 (minor coat protein, CPm), p25 (major coat protein, CP), p18, p13, p20, and p23 [4,11,12]. The major coat protein, approximately 25 kDa, encapsulates around 95% of the genome, whereas the minor coat protein, around 27 kDa, encapsulates the remaining portion of the genome and is positioned at one extremity of the capsid [13,14]. CTV is primarily transmitted by aphids in a semipersistent manner, and the brown citrus aphid (*Toxoptera citricida Kirkaldy*) has been identified as the most efficient vector [15,16]. The propagation of CTV via the exchange of infected grafts and saplings, coupled with aphid transmission, has resulted in local outbreaks in citrus-producing regions [17,18,19,20]. The brown citrus aphid can transmit CTV within a matter of a few seconds to 30 min. The aphid remains infectious for 24 h and ceases to become infectious after 48 h [21]. This aphid is regulated by Council Directive 2000/29/EC of the European Union (EU), detailing the relevant biological and morphological identification methods, and it is listed as a harmful organism in Annex IIAI, prohibiting its introduction into and spread in the EU [22].

Since the initial characterization of CTV, various methods for detecting and identifying CTV isolates have been developed. These methods include bioassay-based approaches, serological assays, and nucleic acid-based detection techniques [13,23,24,25]. Traditionally, the detection and classification of CTV have relied on biological indices in the greenhouse, particularly the inoculation of indicator plants, such as Mexican limes, sour oranges, sweet oranges, and grapefruits grafted onto sour orange rootstocks [23,26,27]. The European Food Safety Authority (EFSA) stated, in its opinion on CTV, that appropriate biological assays are strictly necessary and that the value of molecular and serological data in determining the genetic and pathogenic characteristics of CTV isolates is limited [27]. Additionally, the EPPO’s guidelines regarding CTV indicate that when CTV is first diagnosed, or in severe cases (import/export), two different screening methods should be used in combination, based on biological detection and serological or molecular detection [26]. This approach provides intuitive and reliable data without the need for instrumental detection. Despite its limitations, including a lengthy observation period and a large field nursery, incurring high human management costs, and challenges in high-throughput detection, this method is necessary for identifying the pathogenic characteristics of CTV.

## 3. Immunological Methods

Immunological or serological methods leveraging specific antibodies are widely used for plant pathogen detection. These methods for CTV detection adopt specific antibody-based technologies, including enzyme-linked immunosorbent assay (ELISA), direct tissue blot immunoassay (DTBIA), lateral flow immunoassay (LFIA), and various serological variants (Table 1), facilitating the convenient, rapid, and low-cost detection of CTV. These methods utilize antibodies that specifically bind to epitopes on antigens [28,29,30]. To detect CTV, the application of enzyme-conjugated, fluorophore-conjugated, or nanoparticle-conjugated specific antibodies has proven effective [31,32,33]. Recent advancements in CTV particle preparation and purification have facilitated the production of polyclonal antibodies (pAbs), thus enhancing CTV detection via electron microscopy (EM) and SDS immunodiffusion [31]. The pAbs capable of recognizing various epitopes on a single antigen [34] are cost effective; however, they suffer from limited shelf life and batch variability. Moreover, antisera may exhibit cross-reactivity with closely related viruses. To minimize these negative impacts, monoclonal antibodies (mAbs) are constructed through the utilization of purified recombinant CTV coat protein (CTV-CP) [31,35,36,37]. In comparison, mAbs offer enhanced batch consistency and specificity, effectively increasing test specificity and reproducibility [38]. However, they incur higher costs, are impacted by reduced sensitivity, and are limited to identifying a single epitope [39,40].

### 3.1. ELISA

ELISA has been the leading immunological method for detecting microbial pathogens since the 1970s. Its rapidity, ability to be automated, and capability for high-throughput screening make it the method of choice across a wide range of applications [38,39,42]. These protocols adopt enzyme-linked antibodies to convert colorless substrates into colored products, enabling the direct quantification of antigens and, thereby, the determination of pathogen load in samples [43,44]. Despite its utility, the ELISA technique is challenged by antibody instability, which requires refrigeration to remedy, and the elaborate and expensive process of producing new antibodies [45]. ELISA is extensively utilized in agriculture for detecting plant pathogens, with its use for CTV detection is notably prevalent compared to other methods [3]. Various ELISA protocols, such as direct antigen-coated ELISA (DAC-ELISA), double antibody sandwich ELISA (DAS-ELISA), and triple antibody sandwich ELISA (TAS-ELISA), have been developed for CTV detection [33,46,47].

CTV detection in citrus trees across 15 diverse altitudinal regions in India was facilitated by the use of CTV pAbs in conjunction with DAC-ELISA. This method effectively identified CTV in symptomatic Kagzi lime plants and confirmed the presence of long, flexuous CTV particles in plants through EM, thereby demonstrating the efficacy of DAC-ELISA in diagnosing CTV infections in trees [48]. Furthermore, an incidence rate of 26.3% for CTV was recorded in the Vidarbha region, adopting both DAC-ELISA and reverse transcription polymerase chain reaction (RT-PCR), thereby confirming the applicability of these methods for epidemiological research on CTV [49]. PAbs derived from purified recombinant CTV-CP exhibited significant reactivity against both homologous and heterologous CTV isolates in DAC-ELISA analyses. Notably, these antisera demonstrated higher sensitivity to CTV isolates from Pakistan compared to commercial DAS-ELISA kits [5]. Therefore, the sensitivity of commercial detection kits requires further improvement.

Initial analyses have documented the development of DAS-ELISA for detecting CTV, utilizing pAbs against bacterially expressed CTV-CP [50]. Subsequently, immunization of BALB/c mice with purified viral particles from the CTV T-308 strain resulted in the production of 3DF1 and 3CA5 mAbs [51]. These antibodies facilitated the development of an optimized DAS-ELISA technique, capable of detecting all CTV isolates from various countries [33]. Additionally, a commercial kit developed by INGENASA, adopting bioluminescent avidin–streptavidin ELISA and a double-antibody sandwich technique, utilized a mixture of CTV-specific monoclonal antibodies, 3DF1 and 3CA5, for antigen detection [52]. In Spain, over two million ELISA tests have been performed in both governmental and private facilities, among which DAS-ELISA is included [53].

The sensitivity of certain ELISAs for CTV detection has been evaluated using both monoclonal and polyclonal antibodies. The TAS-ELISA method is widely adopted due to its high sensitivity and capability of detecting viral proteins at concentrations as low as 0.1 ng/mL [54]. Moreover, companies such as Agdia, Bioreb, and Sanofi also market commercial kits for CTV identification. These kits have demonstrated effectiveness in distinguishing between CTV-positive and -negative samples [46,55]. Four mAbs have been produced, 14B10, 14H11, 20D5, and 20G12, all of which detect the virus in infected leaf tissue crude extracts diluted to 1:10,240 (*w*/*v*, g/mL), indicating that these mAbs and the established TAS-ELISA are very sensitive for CTV detection [56]. The adoption of engineered antibodies has led to successful applications in diagnosing plant CTV through DTBIA and DAS-ELISA, showcasing high specificity and sensitivity [9]. This example marks a significant advancement in the routine detection of woody plant viruses using recombinant antibodies. Research into recombinant single-chain variable fragment antibodies (SCFV) 3DF1scFv and 3CA5scFv demonstrates their specific binding to CTV, highlighting the potential of such antibody combinations in TAS-ELISA [57]. In addition, scFvF10 was produced by adopting phage display technology, achieving a sensitivity of 0.01 µg/mL in detecting the CTV-CP protein via TAS-ELISA.

Advancements in the technology used to detect CTV antibodies, coupled with the increasing variety of antibodies, have significantly contributed to the epidemiological investigation and prevention of CTV. MCA13 is a unique monoclonal antibody that targets CTV, specifically identifying strains implicated in the severe symptoms observed in affected plants [58]. In contrast, other monoclonal antibodies, including ECTV-175, ECTV-176, 11B1-3, and 3E10-6, demonstrate reactivity with all CTV isolates through both direct and indirect ELISA assays [59]. Conversely, MCA-13 demonstrated strong reactivity to the Cyprus isolate and mild positive reactions to the Igdir-1 and Igdir-2 isolates; however, it failed to react to additional isolates [59]. The response from MCA13 does not correlate directly with the virulence of strains, rendering it unsuitable for distinguishing between samples affected by SP, decline strains, or their combinations [23]. Thus, comprehensive detection necessitates the concurrent use of 3DF1 and 3CA5 monoclonal antibody mixtures or 3E10-6 or 17G11 monoclonal antibodies [26,60,61,62,63]. Furthermore, both severe (MCA-13 response) and mild strains can be detected within the same plant [64]. Subsequent verification of isolates from Spain, the United States, Japan, and other regions have confirmed that the reaction of MCA-13 is determined by the presence of phenylalanine (F) at position 124 of the CP protein amino acid sequence; in contrast, in mild strains, the amino acid at position 124 of the CP protein is tyrosine (Y) [26,65,66]. An increasing number of CTV antibodies have been reported; however, there is still room for improvement in the sensitivity and efficacy of commercial ELISA kits. Additionally, detection devices can be optimized to further reduce costs, thereby making the method more widely accessible.

### 3.2. DTBIA

DTBIA provides a straightforward approach for detecting CTV on nitrocellulose membranes using plant material, facilitating large-scale, on-site sample analysis without the need for complex extraction preparations [61,67,68]. DTBIA is a rapid, sensitive, and reliable method that requires little sample preparation. Tissue prints can be preserved for several months at room temperature (18–25 °C) in a dry, dark environment, allowing for safe transportation to laboratories for analysis [9,69]. Utilizing monoclonal or recombinant antibodies, DTBIA presents a cost-effective and reliable alternative to molecular and other serological assays for the detection of CTV. It has received official endorsements from the EU. In analyses, both ELISA and DTBIA were used to test 858 trees in Florida (both healthy and CTV infected) in addition to 560 trees in Spain, revealing comparable CTV infection rates [61]. Furthermore, adopting MCA13 monoclonal antibodies on the positive specimens expedited the identification of severe CTV strains [61]. DTBIA, popular among nurseries for testing over 500,000 samples at once, since 1994 in Spain, is notable for its simple operation and affordability (0.24 euros per plant), making it suitable for use by non-professionals during large-scale surveys [41]. Remarkably, even a small team of two can process up to 1250 plant tissues or aphids daily [41]. A detection system comprising dot-ELISA, TAS-ELISA, and DTBIA has been developed by expressing the CP protein in *Escherichia coli* and preparing the 14B10 monoclonal antibody [56]. This system confirmed CTV’s widespread presence in citrus trees across the provinces of Chongqing, Jiangxi, and Zhejiang in China, demonstrating over 99.5% concordance between serological and RT-PCR detections [56]. However, through visual colorimetric results, it is difficult to distinguish low-content CTV samples with the naked eye. Therefore, higher sensitivity antibodies or high-sensitivity detection equipment are needed for detection.

### 3.3. LFIA

LFIA represents a leading method for the detection of plant pathogens, demonstrating a strong correlation with ELISA for CTV detection across both leaf and fruit samples upon comparative analysis [70,71]. Accordingly, LFIA for the detection of CTV has been developed, which can detect CTV within 10 min and is still sensitive to crude plant extract diluted 1:80 [32]. LFIA was validated using CTV strains from greenhouse and field sources (T30, T36, VT, and RB), and the results showed 100% consistency with ELISA and RT-PCR [32]. An LFIA test strip provides a cost-effective tool for non-laboratory personnel to detect CTV in the field or in a nursery without laboratory equipment. These test reagents usually have a long shelf life, ensuring stability at room temperature for 12–24 months [72]. These forms of detection aim to detect target pathogens qualitatively, and the results of such tests are provided by visual observation of the color lines in the test area. However, their sensitivity is limited because only a limited number of samples can be loaded into the sample application area [73], and this method is confined to liquid samples. For the detection of solid samples or complex matrices (such as citrus materials), a sample pre-processing step is required during LFIA to extract the relevant target antigens [42]. The processing methods of citrus samples are expected to further optimize in the future, making the operation more simplified and ensuring effective extraction of CTV. Moreover, the use of nanomaterials with high detection sensitivity (such as quantum dots, silver nanoparticles, polymer nanoparticles, etc.) and antibodies will enable the accurate detection of CTV even at low concentrations.

### 3.4. Immuno-Electron Microscopy

The initial observation of inclusion within the phloem tissue of CVT-infected citrus was documented by Schneider and Henry [74]. Subsequently, detection techniques were refined, facilitating rapid and reliable CTV diagnosis through EM, scanning EM, and optical microscopy [75]. Investigations utilizing scanning EM highlighted an association between outgrowths of the phloem tissue and the occurrence of SP. Moreover, a higher presence of viral inclusions was observed in the phloem compared to the other sieve elements [76]. Confocal microscopy analysis revealed a striking difference in infection foci between two citrus species, alemow and sour orange [77]. The more susceptible species exhibited infection foci consisting of multiple cell clusters; in comparison, the less susceptible species showed single-cell infection foci, suggesting the absence of cell-to-cell movement. The results of analyses using immuno-EM suggested that the CTV-MCA13 strain might interact with a hidden epitope on the viral capsid protein [58]. Additionally, the results of immuno-EM showed that in the CTV strains from Florida and Israel, the p27 antibody modified a 75–85 nm segment at one end of the viral particle, and the CP antibody modified most of the viral particles but did not modify the 75–85 nm long terminal segment [13]. This method, compared with other detection methods, allows for a more intuitive observation of the morphology of virus particles and lesions. However, it may require higher costs and is difficult to achieve high-throughput detection of CTV samples.

## 4. Nucleic Acid-Based Detection Methods

Nucleic acid (RNA) sequences serve as exemplary molecular markers for detecting and identifying CTV. RT-PCR, tissue-print real-time RT-PCR (TP-RT-PCR), reverse transcription–loop-mediated isothermal amplification (RT-LAMP), and other amplification and hybridization-based techniques enable the identification of gene sequences characteristic of different CTV genotypes. Compared to ELISA and hybridization methods, RT-PCR exhibits higher sensitivity and is the most practical technique for plant virus detection [30]. PCR surpasses immunoassays with its ability to rapidly and precisely identify multiple targets within complex mixtures, facilitating differentiation between CTV strains through unique PCR primer or probe designs, which is a challenge for serological assays. Crucially, nucleic acid assay testing enables the quantification of CTV levels. The molecular detection of CTV involves the adoption of RNA-based techniques, including an array of PCR variations such as RT-LAMP, reverse transcription droplet digital PCR (RT-ddPCR), and TP-RT-PCR (Table 2), providing rapid, precise, and quantitative detection of CTV.

### 4.1. Conventional RT-PCR/RT-qPCR

The fidelity of DNA hybridization and replication is crucial for widespread viral plant pathogens, including CTV. RT-PCR provides substantial advantages in sensitivity, facilitating the rapid identification of CTV within a few hours [78,79]. Conventional PCR (cPCR) detection methods, despite their high sensitivity and specificity, have several limitations: they require agarose gel electrophoresis for the analysis of the results, which extends the duration of analysis; the need to open tubes during gel electrophoresis increases the risk of contamination; they are more vulnerable to PCR inhibitors, leading to potential false negatives; and require specialized laboratory equipment, restricting use in field conditions; they cannot quantify the target pathogen; and their high sensitivity can facilitate false positives due to the nonspecific amplification of cross-contamination [80,81,82]. Conversely, quantitative RT-PCR (RT-qPCR) provides a more advantageous option, exhibiting higher sensitivity without requiring the traditional post-PCR agarose gel detection step [83,84]. However, compared to conventional RT-PCR, RT-qPCR is more expensive in terms of the required equipment and per reaction cost. Additionally, it involves more complex calculations, making personnel training more challenging.

RT-qPCR techniques utilizing SYBR Green and probe-based fluorescence technology enable the precise quantification of CTV RNA copies in infected citrus tissues or aphids harboring the virus. Nevertheless, the binding of SYBR Green to nonspecific products necessitates additional measures for validation, such as qPCR melt curve analysis [78,85,86,87,88]. Notably, it is possible to quantify sample DNA concentrations through the use of a standard curve, which is critical for confirming the presence of the target CTV, with this approach utilizing known DNA template concentrations to accurately calculate Ct values [83,84,85,86]. A general primer set design, based on the conserved ORF sequences 1b and 2, has been developed to establish an SYBR Green RT-qPCR technique for the quantitative detection of CTV in different tissues of infected plants [85]. Subsequent to the conservative analysis of the CP gene, primers were developed for an SYBR Green fluorescent dye-based real-time RT-PCR assay to precisely quantify CTV titers in the brown citrus aphid during the acquisition access period (AAP) [89].

**Table 2:** Comparison of current nucleic acid-based detection methods for CTV.

Type	Advantages	Limitations	Sample Type	Gene Targeted	Characteristics	References
RT-qPCR	Commercially available More sensitive than cPCR Quantitative analyses	Sophisticated equipment is required Tedious procedure Comparatively higher cost	Total RNA	ORFs 1b and ORFs 2; CP	Quantitatively analyzes CTV content.	[85,86]
TP-RT-PCR	No need to extract nucleic acids Convenient for storage and transportation Removes inhibitors efficiently	Sophisticated equipment is required Low RNA content limits its sensitivity	Pieces of membrane harboring the printed samples	3′ UTR and CP	IPPC-FAO standard recommendations.	[69]
Multiplex RT-PCR	Distinguish co-infections Low cost	Sophisticated equipment is required Susceptible to non-specific amplification	Total RNA	5′UTR, ORF1a, ORF1b, p33, p20, and p23.	Six genotypes were distinguished.	[90]
Nested RT-PCR	No need to extract nucleic acids Low cost	Lower sensitivity than real-time RT-PCR and DAS-ELISA	Crude leaf sap	3′UTR	The sensitivity higher than cPCR, but lower than RT-qPCR and DAS-ELISA.	[78]
RT-ddPCR	Higher accuracy than RT-qPCR Without need for a standard curve Increased tolerance to inhibitors	Limited upper limits may lead to signal saturation Comparatively higher cost	Total RNA	CP	100-fold greater sensitivity than RTqPCR.	[91]
RT-LAMP	No need to extract nucleic acids The detection time is short Low requirements for instruments Result visualization	Difficult to quantify Complex primer design	Total RNA	CP	The minimum amplification time was 6:45 (min:s)	[92]
SSCP	Low requirements for instruments Low cost	Difficult to quantify Limited analytical ability	Total RNA	CP	Screening of mild strains for cross-protection	[93]

Techniques used in CTV nucleic acid-based detection methods testing. RT-qPCR: reverse transcription quantitative PCR, TP-RT-PCR: tissue-print real-time RT-PCR, nested RT-PCR: internal control reverse transcription nested PCR, RT-ddPCR: reverse transcription droplet digital PCR, RT-LAMP: reverse transcription–loop-mediated isothermal amplification, SSCP: single-strand conformation polymorphism analysis. RNA extraction methods reduce preparation time and steps, thereby enhancing overall detection efficiency. Introducing fully automated technology can reduce manual operations and errors, and increase throughput. Additionally, developing portable RT-qPCR devices makes rapid field or on-site detection possible, enabling timely implementation of control measures.

The utilization of quenching probes significantly improves the reliability and specificity of CTV pathogen detection in PCR analyses, offering an advanced level of specificity by ensuring that fluorescence emission only occurs upon the successful hybridization of the probe with its target sequence [86,94,95]. These probes enable the detection of CTV with higher sensitivity and efficiency than ELISA or conventional RT-PCR methods, presenting notable advancements in diagnostics [86]. In detecting RNA levels of CTV, RNA copies as minimal as less than 1 fg are discernible in extracts from infected plants, with aphid-based RNA copy numbers ranging from 12,000 to 13,000,000 [86]. However, this method incurs additional costs compared to alternatives such as SYBR Green [87,88]. Due to its high sensitivity, RT-qPCR is widely used for the detection of CTV. However, the sample preparation is cumbersome, necessitating the development of faster and simpler RNA extraction methods to reduce preparation time and steps, thereby enhancing overall detection efficiency. Introducing fully automated technology can reduce manual operations and errors, and increase throughput. Additionally, developing portable RT-qPCR devices makes rapid field or on-site detection possible, enabling the timely implementation of control measures.

### 4.2. TP-RT-PCR

The IPPC-FAO standard advocates for TaqMan-based real-time RT-PCR diagnosis, detection, and identification of CTV; immobilizing targets from fresh plant tissue onto substrates such as paper, nitrocellulose, or positively charged nylon membranes using printing or pressing techniques is recommended [69]. By using TP-RT-PCR to detect tissue-printed materials, the CTV copy number can be detected, ranging from 1.9 × 10^4^ to 3.7 × 10^6^ copies, and the CTV copy number in single squashed aphids ranges from 4.73 × 10^3^ to 1.23 × 10^5^ copies [78]. Compared to DTBIA, TP-RT-PCR is more sensitive, while DTBIA shows higher specificity, showing that TP-RT-PCR can be used for routine CTV detection [96]. This approach is rapid and biosecure, circumventing quarantine regulations by eliminating the risk of introducing foreign CTV isolates or vectors, and it streamlines sample preparation without necessitating nucleic acid purification, making it ideal for epidemiological analyses or broad screenings. However, challenges remain, such as the instability of the RNA’s structure affecting storage duration under varying conditions and the need to maintain sufficient sensitivity for low RNA levels. Given the significant impact of operational techniques on sensitivity and cross-contamination, it is essential that operators possess a background in molecular biology and receive rigorous training to enhance their skills. The limited RNA content restricts the sensitivity of the current methods, necessitating the development of more sensitive detection reagents and techniques to further reduce false positive and false negative rates. Moreover, employing multiplex PCR technology to detect multiple target genes simultaneously can significantly improve detection efficiency.

### 4.3. Multiplex RT-PCR

Multiplex PCR utilizes multiple primer sets to target diverse gene sequences within a single reaction, facilitating the concurrent detection of various pathogens and CTV strains. This method is a resource-efficient technique that saves time and reduces labor. However, it tends to be more vulnerable to non-specific DNA amplification, which increases the possibility of false positive results [97]. For citrus plants, several multiplex RT-PCR methods have been developed for detecting CTV and other pathogens: a validated method for *Citrus Psorosis Virus* (CPsV) and *Citrus leaf blotch virus* (CLBV) using three fluorescently labeled probes in RT-qPCR, reaching similar limits of detection and sensitivity [98]; an RT-qPCR approach for CPsV and *Citrus Variegation Virus* (CVV) adopting specific primers and TaqMan probes, which maintains sensitivity equivalent to single-target assays [99]; and a technique for simultaneously detecting four pathogens—*Candidatus* Liberibacter asiaticus (CLas), CTV, *citrus tatter leaf virus* (CTLV), *citrus exocortis viroid* (CEVd)—showing exceptional efficacy in field samples [100,101]. Additionally, a one-step multiplex RT-PCR technique that differentiates CTV strains and detects CLas, *Hop Stunt Viroid* (HSVd), and *Citrus Exocortis Viroid* (CEVd) was validated for its sensitivity and efficiency [102]. To distinguish between the various CTV strains, specific primers and probes targeting the coat protein region and the 5′ end of the T36 strain have been designed, as well as the ORF 1a and ORF 2 regions of the T36, T30, and VT strains, and this approach facilitates the relative quantitative analysis of these CTV strains in aphids [103]. Furthermore, a sequencing-based approach utilizing multiplex RT-PCR and microarray hybridization in a lab-on-a-chip (LoC) device has been established, featuring 12 primers and 44 probes targeting six genes [90]. This innovative system quickly discerns genotypic diversity and differentiates between local and non-local isolates through the hybridization patterns of multiple probes, ensuring accurate strain identification. Compared to single RT-PCR, multiplex PCR is more prone to nonspecific amplification. Advanced computational tools and bioinformatics methods are essential for improving the efficiency and accuracy of primer design. Additionally, precise optimization of reaction conditions is crucial to ensure the effective amplification of all target genes under uniform conditions.

### 4.4. Nested RT-PCR

Nested RT-PCR (NRT-PCR) represents an advanced variant of RT-PCR that enhances specificity and sensitivity through two sequential amplification stages. In the first stage, external primers target either the conserved CP gene or the 3′UTR region of CTV to amplify a larger DNA region [104,105]. This PCR product then becomes the template for a second amplification using internal primers that bind to regions amplified in the first stage. Despite requiring more PCR cycles and thus being more prone to carry-over contamination, this method markedly improves the detection sensitivity, especially for low CTV levels [105]. Additionally, the dual PCR process renders NRT-PCR more costly. The introduction of the IC-RT-nested PCR detection technique allows for high-sensitivity CTV detection without requiring nucleic acid purification steps [104]. However, conventional real-time RT-PCR outperforms IC-RT-nested PCR by 100–500 times in sensitivity and is a million times more sensitive than DAS-ELISA [78]. When detecting samples with low CTV content, nested RT-PCR is an excellent choice, as the two consecutive PCR reaction steps significantly enhance sensitivity. However, this also increases the complexity and duration of the experiment, as well as the risk of cross-contamination. Therefore, developing a “one-step” nested RT-PCR procedure, which integrates and automates the process to reduce operational steps and time, can lower the risk of cross-contamination.

### 4.5. Digital Droplet PCR

Digital droplet PCR (ddPCR) builds upon the foundational principles of traditional PCR by introducing precise absolute quantification of nucleic acids in a sample [106]. The method involves partitioning the sample’s DNA across numerous small water–oil droplets, with each droplet potentially containing 0 or 1 DNA molecule template, ddPCR ensures that each droplet acts as an individual PCR reaction chamber. When assessing CTV RNA transcripts through serial dilutions, comparisons were made regarding quantitative linearity, sensitivity, and accuracy between RT-ddPCR and RT-qPCR. Both techniques maintained a strong linear quantitative relationship; however, RT-ddPCR outperformed RT-qPCR in sensitivity by 100-fold and showcased a higher accuracy [91]. In contrast to RT-qPCR, RT-ddPCR does not require calibration curves for quantification, and the DNA present is quantified in a direct manner. The quantification approach of ddPCR enhances reliability by eliminating discrepancies that may arise from varying amplification efficiencies during calibration curve establishment [107,108]. The detection limit of the RT-ddPCR was 11.4 copies by RT-ddPCR, and an amplification time of only 10 min and 35 s [92]. Furthermore, ddPCR exhibits increased sensitivity and heightened tolerance to inhibitors compared to qPCR. At present, the ddPCR equipment and reagents required for such experiments are expensive, and few laboratories and research institutions can afford them [109]. The cost of ddPCR testing is twice that of qPCR, and ddPCR equipment is more expensive than qPCR equipment [110]. In the future, technological innovation and mass production may reduce the cost of ddPCR equipment and consumables, enabling its application in more laboratories. Additionally, developing more convenient sample preparation and droplet generation methods, along with high-throughput ddPCR platforms, may reduce the operational steps, allowing more CTV samples to be processed in a shorter time and increasing overall detection throughput.

### 4.6. RT-LAMP

LAMP has emerged as a novel gene amplification technique that functions as an effective and rapid diagnostic tool for the early detection of CTV nucleic acid targets. Its benefits include the ability to conduct LAMP reactions in cost-effective, simple isothermal conditions, such as a water bath or dry bath at 60–65 °C [111] and the use of SYBR Green for result observation via color changes, eliminating the need for additional equipment [112]. Moreover, its tolerance to PCR inhibitors allows the use of crude extracts, leading to reduced analysis time and cost. Furthermore, LAMP has facilitated the detection of CTV through the use of four primer sets targeting the CP gene, with results visible to the naked eye or on a 1.5% agarose gel [111]. The sensitivity of the CTV-RT-LAMP protocol was found to be 100 times greater than that of the conventional one-step RT-PCR assay [112]. CTV-RT-LAMP showcases the potential for application in remote locations with a sensitivity of 0.002 ng RNA [92]. The development of immunocapture RT-LAMP (IC-RT-LAMP) further enhances rapid on-site detection by capturing CTV virions directly from crude citrus leaf sap, eliminating the nucleic acid extraction step and achieving a minimum amplification time of 6 min and 45 s [92]. However, LAMP’s primary limitations include its difficulty in quantitative analysis and the complexity of primer design [113], and suboptimal primers can lead to the formation of nonspecific products and primer dimers. Given the challenge in distinguishing between fluorescence signals generated by specific versus nonspecific products, this situation may give rise to false positive outcomes. Advanced bioinformatics tools and algorithms to optimize primer design and reaction conditions should be utilized, to reduce nonspecific amplification. New real-time monitoring technologies and protocols need to be developed to enable RT-LAMP to provide accurate quantitative results for CTV similar to RT-qPCR, as well as the establishment of standard operating procedures (SOPs) and quality control standards to ensure the comparability of RT-LAMP detection results across different laboratories. More sensitive visualization methods, such as using high-sensitivity fluorescent probes or nanomaterials, to improve the accuracy of visual observation need to be developed.

### 4.7. Single-Strand Conformation Polymorphism Analysis (SSCP)/Capillary Electrophoresis SSCP (CE-SSCP)

The SSCP method is based on the ability of single-stranded DNA to form unique conformations under non-denaturing conditions according to its primary sequence. Its structure is stabilized by intramolecular interactions and has been widely used to detect genetic variations and identify plant pathogens. Using the SSCP of the CTV structural protein CP gene, 12 isolates from different regions were analyzed. The results showed that mild and severe strains exhibited different corresponding band patterns. This method can serve as an optimal basis for mild strain cross-protection (MSCP) [93]. The SSCP detection method for this gene has been utilized in multiple locations for CTV strain detection to monitor the introduction of new strains and the vector-transmitted characteristics of CTV strains [114,115]. The MCA13 monoclonal antibody can recognize severe strains. Using SSCP analysis on the CP gene of antibody-positive colonies in Colombia, at least seven electrophoretic patterns were identified [116]. Furthermore, the CP gene obtained using immunocapture reverse transcription polymerase chain reaction (IC-RT-PCR) can also be subjected to SSCP analysis to determine whether different haplotypes are present in each isolate [117]. In addition to SSCP analysis of a single CP gene, combined multi-gene analysis is also commonly used. By performing SSCP analysis on the external CP and non-structural p20 genes, it was found that MCA13-reactive positive isolates are related to the T3-like genotype, which can cause severe symptoms in the host [118]. Another structural protein gene, CPm, was subjected to SSCP and sequence analysis, and the nucleotide sequence diversity of the CPm genes in field isolates was evaluated, distinguishing mild pathogenic strains [119]. For non-structural proteins p18, p13, p20, and p23, one to four of these genes were used for the SSP analysis of field plants and aphid-transmitted CTV isolates, monitoring changes in the CTV virus population and cross-protection between strong and weak strains [120].

Capillary electrophoresis (CE) separates molecules with different charges, sizes, or shapes via electrophoresis performed within a capillary tube. Using capillary electrophoresis–single strand conformation polymorphism (CE-SSCP) to analyze the p18, p23, and p27 genes of CTV isolates from Sicily, the feasibility of this method was verified through nucleotide sequence analysis homology [121]. Furthermore, the CP gene is also commonly used in automatic sequencing detection by CE-SSCP [122]. Compared to SSCP, CE-SSCP increases the potential for high-throughput analysis, high resolution, high sensitivity, and reproducibility of results; however, it requires specialized capillary electrophoresis equipment, which is relatively expensive. Additionally, combining CE-SSCP with restriction fragment length polymorphism (RFLP) or multiple molecular markers (MMMs) for identifying CTV strains and mixed isolates demonstrates superior detection performance [123,124,125]. This combination can enhance detection sensitivity and accuracy and aid in the further analysis of the single-strand conformation polymorphism and markers of fragments, providing multi-layered information. However, the experimental design and operation of combined technologies are relatively complex, posing higher technical demands for laboratories. Compared to single CE-SSCP detection, the combined technique requires multiple types of equipment and reagents, resulting in higher costs and increased analysis time. SSCP and CE-SSCP face limitations in detecting low-abundance mutations, potentially missing low-frequency variations in CTV. Employing higher-resolution electrophoresis techniques, such as high-performance capillary electrophoresis (HPCE), can enhance the detection capability for small sequence changes. Variations in experimental parameters, such as temperature and polyacrylamide concentration, can significantly impact the reproducibility and reliability of results. Establishing SOPs can mitigate these issues. The diverse migration rates and conformational changes of different DNA fragments complicate result analysis and interpretation. Advanced data analysis software, which automates the processing and interpretation of complex electrophoresis patterns, could improve the efficiency and accuracy of analyzing results.

## 5. Conclusions

The global citrus trade has experienced rapid growth coupled with the prosperity of the world economy [126]. The long-distance transportation of seedlings, global warming, and the consequent migration of efficient vector species have increased the risk of CTV transmission [127]. Monitoring imported and exported citrus products across regions is crucial, as various CTV strains present distinct symptoms in different citrus varieties, including asymptomatic carriers. Some varieties, acting as asymptomatic carriers, can transmit the virus to other healthy but susceptible citrus varieties via vectors. Although tolerant rootstocks can mitigate the symptoms of rapid decline, a more effective strategy against aggressive CTV strains that cause SP is pre-immunization by asymptomatic or mild strains. This approach leverages the cross-protection phenomenon, in which initial infection by a mild or symptomless strain of the virus can protect plants from more severe strains [4]. Countries such as Brazil, South Africa, and Peru have dedicated years to screening numerous CTV strains for their potential protective capacity; however, these strains often fail to provide protection. In California, evaluations of over 100 viral strains have also failed to identify strains with successful cross-protection against severe strains [128]. Therefore, it is crucial to strengthen nursery management, select appropriate detection methods for CTV, and distinguish between severe and mild strains. Biological assays play an indispensable role in identifying severe and mild strains, as well as CTV-free plants. When combined with serological and molecular diagnostic techniques, these assays are crucial in ensuring the health of seedlings and controlling the spread of disease.

Considering the diverse aspects and complexities of CTV detection, the aim of the present review was to survey the current landscape of CTV detection methods, elaborating on their advantages and limitations, as outlined in Table 1 and Table 2. The biological identification of CTV provides the most direct and reliable evidence through biological indicators in greenhouses. The value of molecular and serological data in determining the genetic and pathogenic characteristics of CTV isolates is limited, so bioassays are strictly necessary. This method is simple and does not require the investment of reagents and equipment, but it has obvious limitations in terms of cost and analysis time. In contrast, immunoassays offer high specificity, rapid analysis, and simple sample preparation, yielding quick results within hours. ELISA kits, in particular, present lower equipment costs and greater applicability compared to other molecular detection methods despite their generally lower sensitivity for CTV detection. When funding is limited, standard laboratory equipment is available, and a large number of CTV samples need to be tested at once, ELISA is an excellent choice. Nucleic acid detection methods for CTV combine high sensitivity and specificity with rapid analysis; however, they require more time for RNA preparation, qualitative analysis, cDNA synthesis, and amplification. These methods often require a clean laboratory setting, extending the overall analysis time to possibly several days. Isothermal amplification techniques address these time concerns effectively and are ideal for detection in citrus trees on-site, requiring only a small amount of tissue preparation under simple isothermal conditions to produce visually discernible results. Nevertheless, such methods, including cPCR and isothermal amplification, fall short of quantitatively assessing CTV content compared to qPCR methods. Recent advancements have introduced high-throughput detection and precise technologies, such as ddPCR, which is distinguished by its elimination of the need for calibration curves in quantification. Although multiplex RT-PCR can detect multiple pathogens simultaneously, it risks false positives due to non-specific DNA amplification. Hybridization techniques and microarrays, which use multiple probes for concurrent pathogen detection, overcome these multiplexing limitations but are more vulnerable to non-specific DNA amplification.

Recent decades have witnessed the emergence of numerous new detection technologies for CTV pathogens. However, it is imperative to rigorously validate the usability, specificity, and sensitivity of these technologies. Furthermore, there is a substantial possibility for additional enhancement of the performance of these technologies. The widespread distribution of CTV in citrus roots, stems, and leaves, coupled with its dynamic spatial and temporal variations in the field, makes preventive monitoring for potential infections arduous, time consuming, and labor intensive. In conclusion, it is evident that an ideal CTV detection method is not yet available. The choice of method is mainly related to the purpose of the application but is limited by the available budget, the type of CTV sample, and the technological resources of the region. However, newly developed technologies are increasingly being applied to CTV disease monitoring.

## Figures and Tables

**Table 1:** Comparison of current serological detection methods for CTV.

Type	Advantages	Limitations	Sample Type	Antibodies	Characteristics	References
ELISA	Commercially available Convenient for large volume detection High sensitivity and specificityLow cost	Sophisticated equipment is required Tedious procedure	Crude leaf sap	MCA13 MCAs, 3DF1 + 3CA5 MCAs	Multiantibody coating and monoclonal antibody were used as primary antibodies for detection	[23]
DTBIA	Convenient for long time storage and long distance transportation Can be tested away from the lab Low cost	Subjective interpretation Sensitivity limitation	Pieces of membranes harboring the printed samples	3DF1 + 3CA5 MCAs linked to AP or 3DF1 scFv-AP/S + 3CA5 scFvAP/S fusion proteins	No antibody coating required, pAbs, monoclonal antibodies or single chain antibodies are used	[41]
LFIA	Easy to carry and use The detection time is short Result visualization Low cost	The sample needs to be pretreated into a liquid Sensitivity limitation	Crude leaf sap	Capture antibody (goat anti-CTV antibody)	Capture antibody was used for detection	[32]
Immuno-electron microscopy (EM)	The virion morphology can be observed Lesions can be observed	Comparatively higher cost Difficult to detect high throughput	Tissue section/purified particles	873, 894 and 879 pAbs	Polyclonal antibody was used for detection	[31]

Techniques used in CTV serological testing. ELISA: enzyme-linked immunosorbent assay, DTBIA: direct tissue blot immunoassay, LFIA: lateral flow immunoassay.

## References

[1] Licciardello G., Scuderi G., Russo M., Bazzano M., Bar-Joseph M., Catara A.F. (2023). Minor Variants of Orf1a, p33, and p23 Genes of VT Strain Citrus Tristeza Virus Isolates Show Symptomless Reactions on Sour Orange and Prevent Superinfection of Severe VT Isolates. Viruses.

[2] Cook G., Breytenbach J.H., Steyn C., de Bruyn R., van Vuuren S.P., Burger J.T., Maree H.J. (2021). Grapefruit field trial evaluation of Citrus tristeza virus T68-strain sources. Plant Dis..

[3] Moreno P., Ambrós S., Albiach-Martí M.R., Guerri J., Pena L. (2008). Citrus tristeza virus: A pathogen that changed the course of the citrus industry. Mol. Plant Pathol..

[4] Folimonova S.Y., Sun Y.-D. (2022). Citrus Tristeza Virus: From Pathogen to Panacea. Annu. Rev. Virol..

[5] Abbas Z., Hameed S., Lockhart B., Bratsch S., Olszewski N., Rehman M., Saqlan Naqvi S. (2021). Production and evaluation of polyclonal antibodies against homologous Citrus tristeza virus (CTV) isolates from Pakistan. JAPS J. Anim. Plant Sci..

[6] Bester R., Cook G., Maree H.J. (2021). Citrus tristeza virus genotype detection using high-throughput sequencing. Viruses.

[7] Folimonova S.Y. (2020). Citrus tristeza virus: A large RNA virus with complex biology turned into a valuable tool for crop protection. PLoS Pathog..

[8] Ayllón M.A., Gowda S., Satyanarayana T., Dawson W.O. (2004). cis-acting elements at opposite ends of the Citrus tristeza virus genome differ in initiation and termination of subgenomic RNAs. Virology.

[9] Iftikhar Y., Abbas M., Mubeen M., Zafar-ul-Hye M., Bakhtawar F., Bashir S., Sajid A., Shabbir M.A. (2021). Overview of Strain Characterization in Relation to Serological and Molecular Detection of Citrus tristeza Closterovirus. Phyton-Int. J. Exp. Bot.

[10] Karasev A., Boyko V., Gowda S., Nikolaeva O., Hilf M., Koonin E., Niblett C., Cline K., Gumpf D., Lee R. (1995). Complete sequence of the citrus tristeza virus RNA genome. Virology.

[11] Dawson W.O., Bar-Joseph M., Garnsey S.M., Moreno P. (2015). Citrus tristeza virus: Making an ally from an enemy. Annu. Rev. Phytopathol..

[12] Sun Y.D., Folimonova S.Y. (2022). Location matters: From changing a presumption about the Citrus tristeza virus tissue tropism to understanding the stem pitting disease. New Phytol..

[13] Febres V., Ashoulin L., Mawassi M., Frank A., Bar-Joseph M., Manjunath K., Lee R., Niblett C. (1996). The p27 protein is present at one end of citrus tristeza virus particles. Phytopathology.

[14] Sekiya M.E., Lawrence S.D., McCaffery M., Cline K. (1991). Molecular cloning and nucleotide sequencing of the coat protein gene of citrus tristeza virus. J. Gen. Virol..

[15] Shangguan C., Kuang Y., Chen Z., Yu X. (2024). Screening candidate effectors from the salivary gland transcriptomes of brown citrus aphid, Aphis citricidus. Arthropod-Plant Interact..

[16] Paiva P.E.B., Neto L.M., Marques N.T., Duarte B.Z., Duarte A.M. (2024). Citrus Aphids in Algarve Region (Portugal): Species, Hosts, and Biological Control. Ecologies.

[17] Korkmaz S., Karanfil A., Satar S., Uslu T., Koç N.K., Çevik B. (2022). Effects of graft and aphid transmission on the genetic diversity and population structure of Turkish citrus tristeza virus isolates. Eur. J. Plant Pathol..

[18] Albertini D., Vogel R., Bové C., Bové J. (1988). Transmission and Preliminary Characterization of Citrus Tristeza Virus Strain. Int. Organ. Citrus Virol. Conf. Proc. (1957–2010).

[19] Chen Y., Yi L., Zhong K., Wang C., Chen B., Li S. (2023). Population genetic characteristics of citrus tristeza virus from wild mandarins in the Nanling Mountains of China. Trop. Plant Pathol..

[20] Roistacher C., Bar-Joseph M. (1987). Aphid transmission of citrus tristeza virus: A review. Phytophylactica.

[21] Bar-Joseph M., Marcus R., Lee R.F. (1989). The continuous challenge of citrus tristeza virus control. Annu. Rev. Phytopathol..

[22] Jeger M., Bragard C., Caffier D., Candresse T., Chatzivassiliou E., Dehnen-Schmutz K., Gilioli G., Grégoire J.-C., Jaques Miret J.A., EFSA Panel on Plant Health (PLH) (2018). Pest categorisation of *Toxoptera citricida*. EFSA J..

[23] Garnsey S.M., Civerolo E.L., Gumpf D.J., Paul C., Hartung J.S. (2005). Biological Characterization of an International Collection of Citrus tristeza virus (CTV) Isolates. Int. Organ. Citrus Virol. Conf. Proc. (1957–2010).

[24] Ruiz-Ruiz S., Moreno P., Guerri J., Ambrós S. (2009). Discrimination between mild and severe Citrus tristeza virus isolates with a rapid and highly specific real-time reverse transcription-polymerase chain reaction method using TaqMan LNA probes. Phytopathology.

[25] Gonsalves D., Purcifull D., Garnsey S. (1978). Purification and serology of citrus tristeza virus. Phytopathology.

[26] Lbida B., Bennani A., Serrhini M.N., Zemzami M. (2005). Biological, serological and molecular characterization of three isolates of Citrus tristeza closterovirus introduced into Morocco. EPPO Bull..

[27] Jeger M., Bragard C., Caffier D., Dehnen-Schmutz K., Gilioli G., Gregoire J.C., Jaques Miret J.A., Macleod A., Navajas Navarro M., Niere B. (2017). Pest categorisation of Citrus tristeza virus (non-European isolates). EFSA J..

[28] López M.M., Bertolini E., Olmos A., Caruso P., Gorris M.T., Llop P., Penyalver R., Cambra M. (2003). Innovative tools for detection of plant pathogenic viruses and bacteria. Int. Microbiol..

[29] Fang Y., Ramasamy R.P. (2015). Current and prospective methods for plant disease detection. Biosensors.

[30] Venbrux M., Crauwels S., Rediers H. (2023). Current and emerging trends in techniques for plant pathogen detection. Front. Plant Sci..

[31] Brlansky R., Garnsey S., Lee R., Purcifull D. (1984). Application of citrus tristeza virus antisera in labeled antibody, immuno-electron microscopical and sodium dodecyl immunodiffusion tests. Int. Organ. Citrus Virol. Conf. Proc. (1957–2010).

[32] Maheshwari Y., Selvaraj V., Hajeri S., Ramadugu C., Keremane M.L., Yokomi R.K. (2017). On-site detection of Citrus tristeza virus (CTV) by lateral flow immunoassay using polyclonal antisera derived from virions produced by a recombinant CTV. Phytoparasitica.

[33] Raeisi H., Safarnejad M.R., Sadeghkhani F. (2022). A new single-chain variable fragment (scFv) antibody provides sensitive and specific detection of citrus tristeza virus. J. Virol. Methods.

[34] Robison B.J. (1995). Use of commercially available ELISA kits for detection of foodborne pathogens. Diagnostic Bacteriology Protocols.

[35] Nikolaeva O.V., Karasev A.V., Garnsey S.M., Lee R.F. (1998). Serological differentiation of the citrus tristeza virus isolates causing stem fitting in sweet orange. Plant Dis..

[36] Nikolaeva O., Karasev A., Powell C., Gumpf D., Garnsey S., Lee R. (1996). Mapping of epitopes for citrus tristeza virus-specific monoclonal antibodies using bacterially expressed coat protein fragments. Phytopathology.

[37] Shokri E., Hosseini M., Sadeghan A.A., Bahmani A., Nasiri N., Hosseinkhani S. (2020). Virus-directed synthesis of emitting copper nanoclusters as an approach to simple tracer preparation for the detection of Citrus Tristeza Virus through the fluorescence anisotropy immunoassay. Sens. Actuators B Chem..

[38] Alvarez A.M. (2004). Integrated approaches for detection of plant pathogenic bacteria and diagnosis of bacterial diseases. Annu. Rev. Phytopathol.

[39] Martinelli F., Scalenghe R., Davino S., Panno S., Scuderi G., Ruisi P., Villa P., Stroppiana D., Boschetti M., Goulart L.R. (2015). Advanced methods of plant disease detection. A review. Agron. Sustain. Dev..

[40] Ascoli C.A., Aggeler B. (2018). Overlooked benefits of using polyclonal antibodies. Biotechniques.

[41] Cambra M., Gorris M., Román M., Terrada E., Garnsey S., Camarasa E., Olmos A., Colomer M. (2000). Routine detection of citrus tristeza virus by direct immunoprinting-ELISA method using specific monoclonal and recombinant antibodies. Int. Organ. Citrus Virol. Conf. Proc. (1957–2010).

[42] Posthuma-Trumpie G.A., Korf J., van Amerongen A. (2009). Lateral flow (immuno) assay: Its strengths, weaknesses, opportunities and threats. A literature survey. Anal. Bioanal. Chem..

[43] Kumar A., Singh U., Kumar J., Garg G. (2008). Application of molecular and immuno-diagnostic tools for detection, surveillance and quarantine regulation of Karnal bunt (Tilletia indica) of wheat. Food Agric. Immunol..

[44] Atmar R.L. (2014). Immunological detection and characterization. Viral Infections of Humans: Epidemiology and Control.

[45] Sakamoto S., Putalun W., Vimolmangkang S., Phoolcharoen W., Shoyama Y., Tanaka H., Morimoto S. (2018). Enzyme-linked immunosorbent assay for the quantitative/qualitative analysis of plant secondary metabolites. J. Nat. Med..

[46] Magdalena Iracheta-Cárdenas M., Alberto Rocha-Peña M. (2005). Comparación de Antisueros Comerciales para la Detección del Virus Tristeza de los Cítricos. Rev. Mex. Fitopatol..

[47] Llanes-Alvarez Y., Peña-Bárzaga I., Batista-Le Riverend L., Pacheco R., Zamora-Rodríguez V., Benítez-Galeano M.J., Rivas F., Bertalmío A., Hernández-Rodríguez L. (2021). Prevalence of mild citrus tristeza virus isolates of the T30 genotype in Cuban commercial citrus fields after the dissemination of huanglongbing. Crop Prot..

[48] Biswas K. (2008). Molecular diagnosis of Citrus tristeza virus in mandarin (Citrus reticulata) orchards of Darjeeling hills of West Bengal. Indian J. Virol..

[49] Biswas K., Tarafdar A., Sharma S., Singh J., Dwivedi S., Biswas K., Jayakumar B. (2014). Current status of Citrus tristeza virus incidence and its spatial distribution in citrus growing geographical zones of India. Indian J. Agric. Sci..

[50] Moreno P., Cambra M., Navarro L., Fernandez Montes J., Pina J.A., Ballester J.F., Juarez J. (1980). A Survey of Citrus Tristeza Virus (CTV) in the Area of Sevilla (Spain) Using the ELISA Method. https://pascal-francis.inist.fr/vibad/index.php?action=getRecordDetail&idt=PASCALAGROLINEINRA8110459313.

[51] Vela C., Cambra M., Cortés E., Moreno P., Miguet J., Pérez de San Román C., Sanz A. (1986). Production and characterization of monoclonal antibodies specific for citrus tristeza virus and their use for diagnosis. J. Gen. Virol..

[52] Cambra M. (2018). Historias de la Inmunología/Serología en España contadas por un fitopatólogo. Fitopatología.

[53] Cambra M., Gorris M.T., Marroquın C., Román M.P., Olmos A., Martınez M.C., de Mendoza A.H., Lopez A., Navarro L. (2000). Incidence and epidemiology of Citrus tristeza virus in the Valencian Community of Spain. Virus Res..

[54] Cambra M., Camarasa E., Gorris M., Garnsey S., Carbonell E. (1991). Comparison of different immunosorbent assays for citrus tristeza virus (CTV) using CTV specific monoclonal and polyclonal antibodies. Int. Organ. Citrus Virol. Conf. Proc. (1957–2010).

[55] Cambra M., Garnsey S., Permar T., Henderson C., Gumpf D., Vela C. (1990). Detection of citrus tristeza virus (CTV) with a mixture of monoclonal antibodies. Phytopathology.

[56] Liu Z., Chen Z., Hong J., Wang X., Zhou C., Zhou X., Wu J. (2016). Monoclonal antibody-based serological methods for detecting Citrus tristeza virus in citrus groves. Virol. Sin..

[57] Terrada E., Kerschbaumer R.J., Giunta G., Galeffi P., Himmler G., Cambra M. (2000). Fully “recombinant enzyme-linked immunosorbent assays” using genetically engineered single-chain antibody fusion proteins for detection of Citrus tristeza virus. Phytopathology.

[58] Permar T., Garnsey S., Gumpf D., Lee R. (1990). A monoclonal antibody that discriminates strains of citrus tristeza virus. Phytopathology.

[59] KORKMAZ S. (2002). Application of direct tissue blot immunoassay in comparison with DAS-ELISA for detection of Turkish isolates of citrus tristeza closterovirus (CTV). Turk. J. Agric. For..

[60] Rocha-Peña M.A., Lee R.F. (1991). Serological techniques for detection of citrus tristeza virus. J. Virol. Methods.

[61] Garnsey S., Permar T., Cambra M., Henderson C. (1993). Direct tissue blot immunoassay (DTBIA) for detection of citrus tristeza virus (CTV). Int. Organ. Citrus Virol. Conf. Proc. (1957–2010).

[62] Powell C., Pelosi R., Rundell P., Stover E., Cohen M. (1999). Cross-protection of grapefruit from decline-inducing isolates of citrus tristeza virus. Plant Dis..

[63] Lin Y., Rundell P.A., Xie L., Powell C.A. (2000). In situ immunoassay for detection of Citrus tristeza virus. Plant Dis..

[64] Cevik B., Pappu S., Pappu H., Benscher D., Irey M., Lee R., Niblett C. (1996). Application of bi-directional PCR to citrus tristeza virus: Detection and strain differentiation. Int. Organ. Citrus Virol. Conf. Proc. (1957–2010).

[65] Kano T., Hiyama T., Natsuaki T., Imanishi N., Okuda S., Ieki H. (1998). Comparative sequence analysis of biologically distinct isolates of citrus tristeza virus in Japan. Jpn. J. Phytopathol..

[66] Pappu H., Pappu S., Manjunath K., Lee R., Niblett C. (1993). Molecular characterization of a structural epitope that is largely conserved among severe isolates of a plant virus. Proc. Natl. Acad. Sci. USA.

[67] Cambra M., Camarasa E., Gorris M., Román M. (1994). Distribución actual de la tristeza de los cítricos y nuevos métodos de diagnóstico. Phytoma.

[68] Cambra M., Gorris M.T., Camarasa E., Román M., Narváez G., Terrada E., Martínez M.C. (1999). Inmunoimpresión-ELISA: Método ideal para detección del virus de la tristeza de los cítricos. Comunitat Valencia. Agrar..

[69] Cambra M., Vidal E., Martinez C., Bertolini E., Catara A.F., BarJoseph M., Licciardello G. (2019). Tissue-Print and Squash Capture Real-Time RT-PCR Method for Direct Detection of Citrus tristeza virus (CTV) in Plant or Vector Tissues. Citrus Tristeza Virus: Methods and Protocols.

[70] Lopez-Soriano P., Noguera P., Gorris M.T., Puchades R., Maquieira A., Marco-Noales E., López M.M. (2017). Lateral flow immunoassay for on-site detection of Xanthomonas arboricola pv. pruni in symptomatic field samples. PLoS ONE.

[71] Salomone A., Mongelli M., Roggero P., Boscia D. (2004). Reliability of detection of Citrus tristeza virus by an immunochromatographic lateral flow assay in comparison with ELISA. J. Plant Pathol..

[72] Ahmed S., Ning J., Peng D., Chen T., Ahmad I., Ali A., Lei Z., Abu bakr Shabbir M., Cheng G., Yuan Z. (2020). Current advances in immunoassays for the detection of antibiotics residues: A review. Food Agric. Immunol..

[73] Koczula K.M., Gallotta A. (2016). Lateral flow assays. Essays Biochem..

[74] Schneider H. (1957). The anatomy of tristeza-virus-infected citrus. Int. Organ. Citrus Virol. Conf. Proc. (1957–2010).

[75] Garnsey S., Christie R., Derrick K., Bar-Joseph M. (1980). Detection of citrus tristeza virus. II. Light and electron microscopy of inclusions and viral particles. Int. Organ. Citrus Virol. Conf. Proc. (1957–2010).

[76] Brlansky R., Howd D., Broadbent P., Damsteegt V. (2002). Histology of sweet orange stem pitting caused by an Australian isolate of Citrus tristeza virus. Plant Dis..

[77] Folimonova S.Y., Folimonov A.S., Tatineni S., Dawson W.O. (2008). Citrus tristeza virus: Survival at the edge of the movement continuum. J. Virol..

[78] Bertolini E., Moreno A., Capote N., Olmos A., de Luis A., Vidal E., Pérez-Panadés J., Cambra M. (2008). Quantitative detection of Citrus tristeza virus in plant tissues and single aphids by real-time RT-PCR. Eur. J. Plant Pathol..

[79] Cook G., van Vuuren S.P., Breytenbach J.H., Burger J.T., Maree H.J. (2016). Expanded strain-specific RT-PCR assay for differential detection of currently known citrus tristeza virus strains: A useful screening tool. J. Phytopathol..

[80] Ward E., Foster S.J., Fraaije B.A., Mccartney H.A. (2004). Plant pathogen diagnostics: Immunological and nucleic acid-based approaches. Ann. Appl. Biol..

[81] López M.M., Llop P., Olmos A., Marco-Noales E., Cambra M., Bertolini E. (2009). Are molecular tools solving the challenges posed by detection of plant pathogenic bacteria and viruses?. Curr. Issues Mol. Biol..

[82] Maurer J.J. (2011). Rapid detection and limitations of molecular techniques. Annu. Rev. Food Sci. Technol..

[83] Singh C., Roy-Chowdhuri S., Luthra R., Singh R.R., Patel K.P. (2016). Quantitative Real-Time PCR: Recent Advances. Clinical Applications of PCR.

[84] Shen C., Shen C. (2019). Chapter 9—Amplification of Nucleic Acids. Diagnostic Molecular Biology.

[85] Ruiz-Ruiz S., Moreno P., Guerri J., Ambrós S. (2007). A real-time RT-PCR assay for detection and absolute quantitation of Citrus tristeza virus in different plant tissues. J. Virol. Methods.

[86] Saponari M., Manjunath K., Yokomi R.K. (2008). Quantitative detection of Citrus tristeza virus in citrus and aphids by real-time reverse transcription-PCR (TaqMan®). J. Virol. Methods.

[87] Mirmajlessi S.M., Loit E., Maend M., Mansouripour S.M. (2015). Real-time PCR applied to study on plant pathogens: Potential applications in diagnosis-a review. Plant Prot. Sci..

[88] Okubara P.A., Schroeder K.L., Paulitz T.C. (2005). Real-time polymerase chain reaction: Applications to studies on soilborne pathogens. Can. J. Plant Pathol..

[89] Liu J., Li L., Zhao H., Zhou Y., Wang H., Li Z., Zhou C. (2019). Titer variation of citrus tristeza virus in aphids at different acquisition access periods and its association with transmission efficiency. Plant Dis..

[90] Scuderi G., Catara A.F., Licciardello G., Catara A.F., BarJoseph M., Licciardello G. (2019). Genotyping Citrus tristeza virus Isolates by Sequential Multiplex RT-PCR and Microarray Hybridization in a Lab-on-Chip Device. Citrus Tristeza Virus: Methods and Protocols.

[91] Wang Y., Yang Z., Zhao J., Li R., Wang Q., Li J., Li Z., Zhou Y. (2020). Development of a sensitive and reliable reverse transcription-droplet digital polymerase chain reaction (RT-ddPCR) assay for the detection of Citrus tristeza virus. Eur. J. Plant Pathol..

[92] Selvaraj V., Maheshwari Y., Hajeri S., Yokomi R. (2019). A rapid detection tool for VT isolates of Citrus tristeza virus by immunocapture-reverse transcriptase loop-mediated isothermal amplification assay. PLoS ONE.

[93] Atta S., Umar U.u.d., Bashir M.A., Hannan A., Rehman A.u., Naqvi S.A.H., Zhou C. (2019). Application of biological and single-strand conformation polymorphism assays for characterizing potential mild isolates of Citrus tristeza virus for cross protection. AMB Express.

[94] Morris T., Robertson B., Gallagher M. (1996). Rapid reverse transcription-PCR detection of hepatitis C virus RNA in serum by using the TaqMan fluorogenic detection system. J. Clin. Microbiol..

[95] Zhou B., Yang G., Hu Z., Chen K., Guo W., Wang X., Du C. (2023). Development of a Real-Time Quantitative PCR Based on a TaqMan-MGB Probe for the Rapid Detection of Theileria haneyi. Microorganisms.

[96] Vidal E., Yokomi R., Moreno A., Bertolini E., Cambra M. (2012). Calculation of diagnostic parameters of advanced serological and molecular tissue-print methods for detection of Citrus tristeza virus: A model for other plant pathogens. Phytopathology.

[97] Lau H.Y., Botella J.R. (2017). Advanced DNA-based point-of-care diagnostic methods for plant diseases detection. Front. Plant Sci..

[98] Osman F., Hodzic E., Kwon S.J., Wang J.B., Vidalakis G. (2015). Development and validation of a multiplex reverse transcription quantitative PCR (RT-qPCR) assay for the rapid detection of Citrus tristeza virus, Citrus psorosis virus, and Citrus leaf blotch virus. J. Virol. Methods.

[99] Loconsole G., Saponari M., Savino V. (2010). Development of real-time PCR based assays for simultaneous and improved detection of citrus viruses. Eur. J. Plant Pathol..

[100] Yao S.M., Wu M.L., Hung T.H. (2023). Development of Multiplex RT-PCR Assay for the Simultaneous Detection of Four Systemic Diseases Infecting Citrus. Agriculture.

[101] Adkar-Purushothama C.R., Quaglino F., Casati P., Bianco P.A. (2010). Reverse transcription-duplex-polymerase chain reaction for simultaneous detection of Citrus tristeza virus and ‘Candidatus Liberibacter’ from citrus plants. J. Plant Dis. Prot..

[102] Saponari M., Loconsole G., Liao H.H., Jiang B., Savino V., Yokomi R.K. (2013). Validation of high-throughput real time polymerase chain reaction assays for simultaneous detection of invasive citrus pathogens. J. Virol. Methods.

[103] Ananthakrishnan G., Venkataprasanna T., Roy A., Brlansky R. (2010). Characterization of the mixture of genotypes of a Citrus tristeza virus isolate by reverse transcription-quantitative real-time PCR. J. Virol. Methods.

[104] Olmos A., Cambra M., Esteban O., Gorris M.T., Terrada E. (1999). New device and method for capture, reverse transcription and nested PCR in a single closed-tube. Nucleic Acids Res..

[105] Adkar-Purushothama C.R., Maheshwar P.K., Sano T., Janardhana G.R. (2011). A Sensitive and Reliable RT-Nested PCR Assay for Detection of Citrus tristeza Virus from Naturally Infected Citrus Plants. Curr. Microbiol.

[106] Hindson B.J., Ness K.D., Masquelier D.A., Belgrader P., Heredia N.J., Makarewicz A.J., Bright I.J., Lucero M.Y., Hiddessen A.L., Legler T.C. (2011). High-throughput droplet digital PCR system for absolute quantitation of DNA copy number. Anal. Chem..

[107] Hayden R., Gu Z., Ingersoll J., Abdul-Ali D., Shi L., Pounds S., Caliendo A. (2013). Comparison of droplet digital PCR to real-time PCR for quantitative detection of cytomegalovirus. J. Clin. Microbiol..

[108] Taylor S.C., Laperriere G., Germain H. (2017). Droplet Digital PCR versus qPCR for gene expression analysis with low abundant targets: From variable nonsense to publication quality data. Sci. Rep..

[109] Lei S., Chen S., Zhong Q. (2021). Digital PCR for accurate quantification of pathogens: Principles, applications, challenges and future prospects. Int. J. Biol. Macromol..

[110] Zhang W., Cui L., Wang Y., Xie Z., Wei Y., Zhu S., Nawaz M., Mak W.-C., Ho H.-P., Gu D. (2023). An Integrated ddPCR Lab-on-a-Disc Device for Rapid Screening of Infectious Diseases. Biosensors.

[111] Ghosh D.K., Warghane A., Biswas K.K., Catara A.F., Bar-Joseph M., Licciardello G. (2019). Rapid and Sensitive Detection of Citrus tristeza virus Using Reverse Transcription Loop-Mediated Isothermal Amplification (RT-LAMP) Assay. Citrus Tristeza Virus: Methods and Protocols.

[112] Warghane A., Misra P., Bhose S., Biswas K.K., Sharma A.K., Reddy M.K., Ghosh D.K. (2017). Development of a simple and rapid reverse transcription-loop mediated isothermal amplification (RT-LAMP) assay for sensitive detection of Citrus tristeza virus. J. Virol. Methods.

[113] Nguyen H.V., Nguyen V.D., Liu F., Seo T.S. (2020). An integrated smartphone-based genetic analyzer for qualitative and quantitative pathogen detection. ACS Omega.

[114] Chatzivassiliou E.K., Nolasco G. (2014). Detection of a new variant of Citrus tristeza virus in Greek citrus crops. Phytopathol. Mediterr..

[115] Zhou Y., Zhou C., Liu K., Liu Y., Wang X. (2011). Influence of the quantity and variability of citrus tristeza virus on transmissibility by single Toxoptera citricida. J. Plant Pathol..

[116] Morales J., Acosta O., Tamayo P., Peñaranda J. (2013). Characterization of Citrus tristeza virus isolates from Colombia. Rev. De Protección Veg..

[117] Silva G., Fonseca F., Santos C., Nolasco G. (2007). Presence of citrus tristeza virus in Angola and São Tomé e Príncipe: Characterization of isolates based on coat protein gene analysis. J. Plant Pathol.

[118] Ferretti L., Fontana A., Sciarroni R., Schimio R., Loconsole G. (2014). Molecular and biological evidence for a severe seedling yellows strain of Citrus tristeza virus spreading in southern Italy. Phytopathol. Mediterr..

[119] Oliveros-Garay O.A., Martinez-Salazar N., Torres-Ruiz Y., Acosta O. (2009). CPm gene diversity in field isolates of Citrus tristeza virus from Colombia. Arch. Virol..

[120] Davino S., Rubio L., Davino M. (2005). Molecular analysis suggests that recent Citrus tristeza virus outbreaks in Italy were originated by at least two independent introductions. Eur. J. Plant Pathol..

[121] Raspagliesi D., Licciardello G., Lombardo G., Catara A., Rizza S. (2009). Quick characterization of Citrus tristeza virus isolates by capillary electrophoresis-single-strand conformation polymorphism. II Int. Symp. Citrus Biotechnol..

[122] Chatzivassiliou E.K., Licciardello G. (2019). Assessment of Genetic Variability of Citrus tristeza virus by SSCP and CE-SSCP. Citrus Tristeza Virus: Methods and Protocols.

[123] Licciardello G., Russo M., Daden M., Bar-Joseph M., Catara A.F. (2015). Capillary Electrophoresis-Single Strand Conformation Polymorphism Analysis and Multiple Molecular Marker Genotyping Allow a Rapid Differentiation of Citrus Tristeza Virus Isolates. Acta Hortic..

[124] Zanutto C.A., Corazza M.J., Nunes W.M.d.C., Müller G.W. (2013). Evaluation of the protective capacity of new mild Citrus tristeza virus (CTV) isolates selected for a preimmunization program. Sci. Agric..

[125] Licciardello G., Xiao C., Russo M., Dai S.M., Daden M., Deng Z.N., Catara A.F. (2015). Genetic Structure of Citrus Tristeza Virus in Hunan Province (P.R. China). Acta Hortic..

[126] Mukhametzyanov R.R., Brusenko S.V., Khezhev A.M., Kelemetov E.M., Kirillova S.S. (2024). Changing the Global Production and Trade of Citrus Fruits. Sustainable Development of the Agrarian Economy Based on Digital Technologies and Smart Innovations.

[127] Černi S., Hančević K., Škorić D. (2020). Citruses in Croatia–cultivation, major virus and viroid threats and challenges. Acta Bot. Croat..

[128] Folimonova S.Y. (2013). Developing an understanding of cross-protection by Citrus tristeza virus. Front. Microbiol..

